# A Robust Method to Detect Zero Velocity for Improved 3D Personal Navigation Using Inertial Sensors

**DOI:** 10.3390/s150407708

**Published:** 2015-03-30

**Authors:** Zhengyi Xu, Jianming Wei, Bo Zhang, Weijun Yang

**Affiliations:** 1 Shanghai Advanced Research Institute, Chinese Academy of Sciences, Shanghai 201210, China; E-Mail: xuzy@sari.ac.cn (Z.X.); zhangbo@sari.ac.cn (B.Z.); yangwj@sari.ac.cn (W.Y.); 2 University of Chinese Academy of Sciences, Shanghai 201210, China

**Keywords:** inertial sensors, personal inertial navigation system, zero velocity detector, bayesian network, kinesiology

## Abstract

This paper proposes a robust zero velocity (ZV) detector algorithm to accurately calculate stationary periods in a gait cycle. The proposed algorithm adopts an effective gait cycle segmentation method and introduces a Bayesian network (BN) model based on the measurements of inertial sensors and kinesiology knowledge to infer the ZV period. During the detected ZV period, an Extended Kalman Filter (EKF) is used to estimate the error states and calibrate the position error. The experiments reveal that the removal rate of ZV false detections by the proposed method increases 80% compared with traditional method at high walking speed. Furthermore, based on the detected ZV, the Personal Inertial Navigation System (PINS) algorithm aided by EKF performs better, especially in the altitude aspect.

## 1. Introduction

As the development of Location Based Services (LBSs), the GPS technique can provide reliable outdoor positioning, but it is invalid in indoor environments. Indoor positioning is required in places like hospitals, warehouses, malls and tunnels. Some commercial indoor location techniques have appeared, such as Wi-Fi fingerprint, RFID, UWB and computer vision, but indoor positioning is still not a well resolved problem so far. All these means need pre-installed infrastructures, network and remote database support. Their high cost also make it impossible to cover all the buildings. On the other hand, when a disaster strikes, the infrastructure becomes fragile. It is very difficult to track emergency first responders by traditional indoor positioning method. Fortunately, with the fast development of Micro Electro Mechanical System (MEMS), it becomes possible to realize reliable personal location for GPS-denied environments using PINS algorithm based on foot-mounted inertial measurement units (IMU). An outstanding advantage of these systems is that they are self-contained, which can compensate for the dead zones of wireless location techniques and provide seamless localization [1,2,3]. This solution is very useful in many applications. For example, tracking emergency first responders [4,5], guiding blind people [6] and augmenting reality. Furthermore, the IMU could be integrated into wearable sensor networks, which could provide rich context information for the future Smart World.

**Figure 1:**
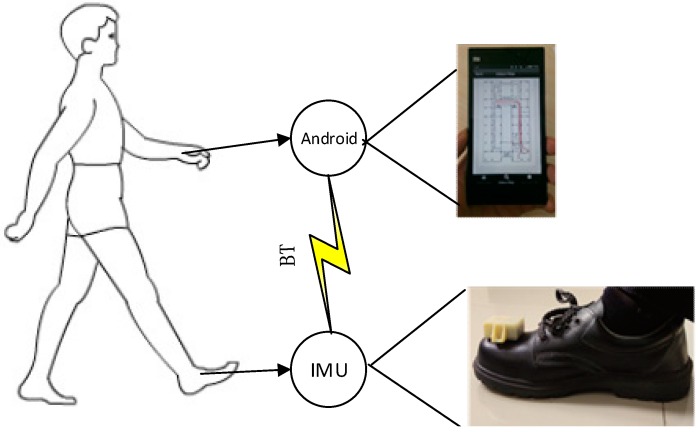
PINS application framework.

The typical PINS application framework is shown in Figure 1. An IMU is mounted on the top of the toe and captures the movement characteristics of the foot. However the irregular movement patterns of people and lack of moving control information like unmanned vehicle systems, make it impossible to track pedestrian position using inertial sensing alone. The position error grows cubically in time brought about by double integrating acceleration errors, which will make the system collapse in the short time. However, during the detected ZV period in a gait cycle, the Zero Velocity Update (ZUPT) and Zero Angular Rate Update (ZARU) features provide pseudo measurements to error states EKF [7,8,9]. This allows the EKF to correct the velocity error and attitude error after each stride, stopping the position error from grow linearly. The reason why ZUPT/ZARU could correct errors is that it tracks the growing correlations among the velocity, position and attitude errors in certain off-diagonal elements of the covariance matrix. For example, at the end of a stride, there exists a high correlation between the uncertainty in the north velocity and the newly accumulated uncertainty in the northing position. If the ZUPT suggested that the velocity error was positive in the north direction, the EKF would correct the position to the south and the velocity to zero.

Since the ZUPT/ZART is so important to PINS, the key problem is how to detect the ZV period accurately, which will directly affect the correction of PINS, especially to the altitude [10]. The traditional threshold method assumes that the acceleration should be only the gravitational acceleration, and the angular rate should be zero during the ZV period, so setting a threshold on the magnitude of the rate-of-turn is applied into a ZV detector [11]. Moreover, a combined condition method is proposed which sets threshold on the acceleration magnitude, local acceleration variance as well as gyroscope magnitude [12], respectively:

(1) $$81<(a_{k,x}^{2}+a_{k,y}^{2}+a_{k,z}^{2})<121$$

(2) $$\frac{1}{n}\sum_{i=1}^{i=j+n}{\left[(a_{i,x}^{2}+a_{i,y}^{2}+a_{i,z}^{2})-\bar{\left(a_{j,x}^{2}+a_{j,y}^{2}+a_{j,z}^{2}\right)}\right]}^{2}<th_{var}$$

(3) $$\omega_{k,x}^{2}+\omega_{k,y}^{2}+\omega_{k,z}^{2}<th_{gyro}$$

where $a_{k,i}$, i=x,y,z is the measurement of an accelerometer in the three orthogonal axes at time k, and $\omega_{k,i}$, i=x,y,z is the gyroscope. Then a logical “AND” allows one to judge ZV by three conditions simultaneously. Finally, the noise in the result is filtered out using a median filter [13]. Based on this method, a dynamic threshold is used to enhance the robustness of the system [14,15]. Furthermore, some probabilistic methods are introduced [16], like the use of the HMM framework to give the probability of movement and standstill for each time instant [17,18]. In order to get a better detection effect, a multi-IMU platform is proposed [19]. Some researchers adopt force sensitive resistors as external zero velocity detectors [20]. These solutions are more reliable, but involve more complex electric designs and higher communication costs.

However, the ZV detectors mentioned above cannot deal with two main problems well. Firstly, the boundaries of the ZV period are fuzzy during the contact period and propulsive period in the gait cycle. Secondly, there exist ZV false detections due to the noise. The proposed solution extends the classic combined condition method, using Bayesian Network method to infer the ZV period with the measurements of low-cost IMUs and kinesiology knowledge. With a high probability of ZV, the method can remove ZV false detections effectively in the midstance period. Aided by the segmentation of the gait cycle, the method could reduce the ambiguity of ZV boundaries. The experiments reveal that the removal rate of ZV false detections increases 80% compared with the classic method at high speed. With the result of this ZV detector, a PINS/EKF aided by ZUPT/ZARU is proposed to realize 3D tracking of pedestrians. The experiments show that the method can achieve good tracking effects and decrease the altitude error by 40% compared with the PINS/EKF based on a combined condition ZV detector.

The remainder of this paper is organized as follows: Section 2 introduces the kinesiology model and proposes a gait cycle detector algorithm. Section 3 details the ZV detector based on Bayesian network inference. Section 4 presents the PINS/EKF framework and Section 5 presents experimental results. Finally, Section 6 provides the conclusions.

## 2. Gait Cycle Detector

**Figure 2:**
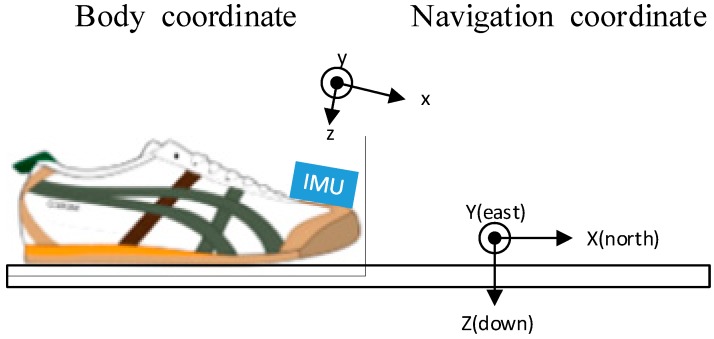
IMU installation mode and the relationship between body coordinate and navigation coordinate.

The basic idea of proposed algorithm is to use an IMU for capturing the movement features of the foot. Different IMU installation modes on the foot create different signal features and ZV periods. To the best of our knowledge, there exist four typical IMU installation modes, namely above the instep, above the toe, under the heel and behind the heel. All of them demand a robust way to accurately detect the ZV period. The key problem is how to reduce the ambiguity at the boundaries of the ZV period. In the experiments, the IMU is mounted on the top of toe (Figure 2). This installation mode has the following advantages:

- The gyroscope measurements in the y axis have a sharp slope at the start of the swing period, which could be easily detected.
- The duration of the ZV period is relative long.
- The IMU measurements are relative stable in the ZV period at high speed.

Figure 2 shows the IMU installation mode and the relationship between body coordinates and navigation coordinates. The following content is analyzed according to this installation mode.

### 2.1. Kinesiology Model Analysis

In order to detect the ZV period in the gait cycle precisely, knowledge of the gait during ambulation is introduced as basic theory. The main content is as follows:

- Gait cycle: a fundamental unit to describe the gait during ambulation, which occurs from the time when the heel of one foot strikes the ground to the time at which the same foot contacts the ground again.
- Heel strike: a heel strike of the same foot.
- A typical gait cycle lasts for 1–2 s.

**Figure 3:**
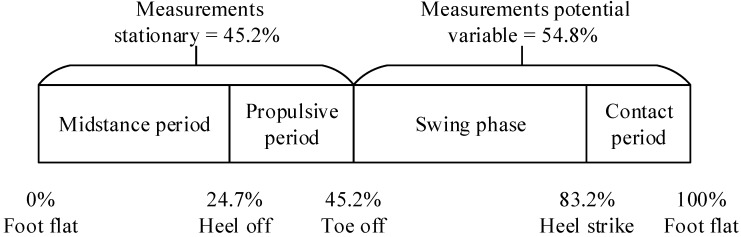
Phases in a typical gait cycle.

According to the kinesiology knowledge, a whole gait cycle consists of the stance phase and swing phase [21] (Figure 3). Moreover, the stance phase includes contact period, midstance period and propulsive period. The IMU signal feature is stationary during the midstance period and propulsive period, but variable during the contact period and swing period. The experiments show that the features of *y* axis gyroscope measurements could be used to segment the gait cycle easily and distinguish the start point of a gait. The gait timespan detector is discussed as follows.

### 2.2. Gait Timespan Calculation

**Figure 4:**
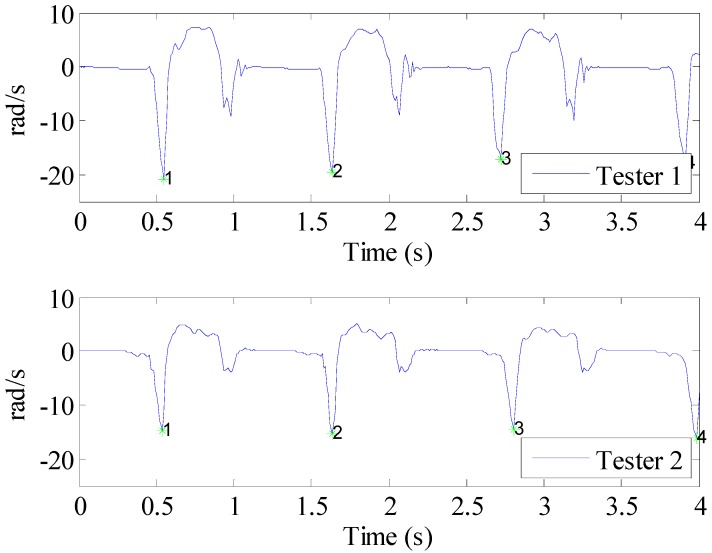
The *y* axis gyroscope measurements of two testers.

In order to design a robust method to calculate gait timespan, the features of IMU measurements should be analyzed first. The proposed solution is based on y axis gyroscope measurements which have a sharp feature at the start of swing phase. Figure 4 shows two typical signal types of testers. The upper subplot shows two valleys in one gait cycle, while in the lower subplot, only one valley could be found with same valley detection algorithm. However, the results of two subplots both show that the same valley could always be found at the beginning of swing period. This valley could be used to calculate the duration of one gait. The details are described as follows:

The well-known zero-derivative method is used to find the valley. Due to the measurement noise or different walking modes, accidental zero-crossings will occur, so some effective means must be adopted based on the signal features of the y axis angular rate. Once the first valley is found, a delay time will be applied to ignore all the other valleys found during this time. This time span is defined according to kinesiology knowledge as:

(4) $$T_{delay}=T_{gait}\times 0.548$$

where $T_{gait}$ is the last gait timespan calculated, 0.548 is the proportion of swing phase and contact period in one gait (Figure 3). Furthermore, a flag is maintained to identify whether the current time instant is in the ZV period. Once the first valley in swing phase is found, the flag is set as false, and if last several samples are ZV, the flag is set as true. When the flag is false, all the other valleys found will also be ignored.

In general, no matter which installation mode is applied, it is inevitable to overcome the ambiguity of the ZV boundary in the contact period. During that short moment, the relative motion between the IMU and foot will create more noise, because the foot strikes the ground. This noise will expand the altitude error, hence, an accurate ZV detector could correct the altitude error better. Based on the kinesiology knowledge and the calculated gait timespan, a data fusion method could be used to reduce the ambiguity of the ZV boundary.

## 3. A Robust Zero Velocity Detector

An accurate ZV detector is an essential part of PINS, because the error states EKF calibrate the position only when the foot is on the ground (ZV). The traditional ZV detectors are based on threshold methods. They could work well for low speed movement, but are not robust enough in some situations. A more reliable ZV detector based on Bayesian network inference is proposed as follows:

### 3.1. Bayesian Inference Model

A Bayesian network is a graphical probabilistic model that represents a set of random variables and their conditional dependencies via a directed acyclic graph. Formally, the nodes represent random variables which may be observable quantities or hypotheses. Edges represent conditional dependencies and nodes that are not connected represent variables that are conditionally independent of each other. Each node is associated with a probability function that takes values for the parent variables, and gives the probability of the variable represented by the node.

When comes to the ZV inference problem, the observable quantities include the sampling time instant, and the IMU measurements. The hypothesis is the proportion of composition in a gait cycle based on kinesiology. The individual observations are correlated. In time-series temporal data, the values of ZV detector are correlated in time and obey the distribution shown in Figure 3. In accelerometer measurement data, the values of the ZV detector are decided by Formula (1). The acceleration measurements of IMU at time k can be written as:

(5) $${\tilde{a}}_{k}=a_{k}+\delta a+e_{k,a}$$

where ${\tilde{a}}_{k}$ is the output of the accelerometer and $a_{k}$ is the real acceleration, δa is the bias, and $e_{k,a}$ is a noise. The threshold in Formula (1) is based on the consideration of the noise. This threshold method could be expanded to use the probability to describe the relationship between ZV and accelerometer measurement. Then a probabilistic graphical model could be used to infer the ZV.

**Figure 5:**
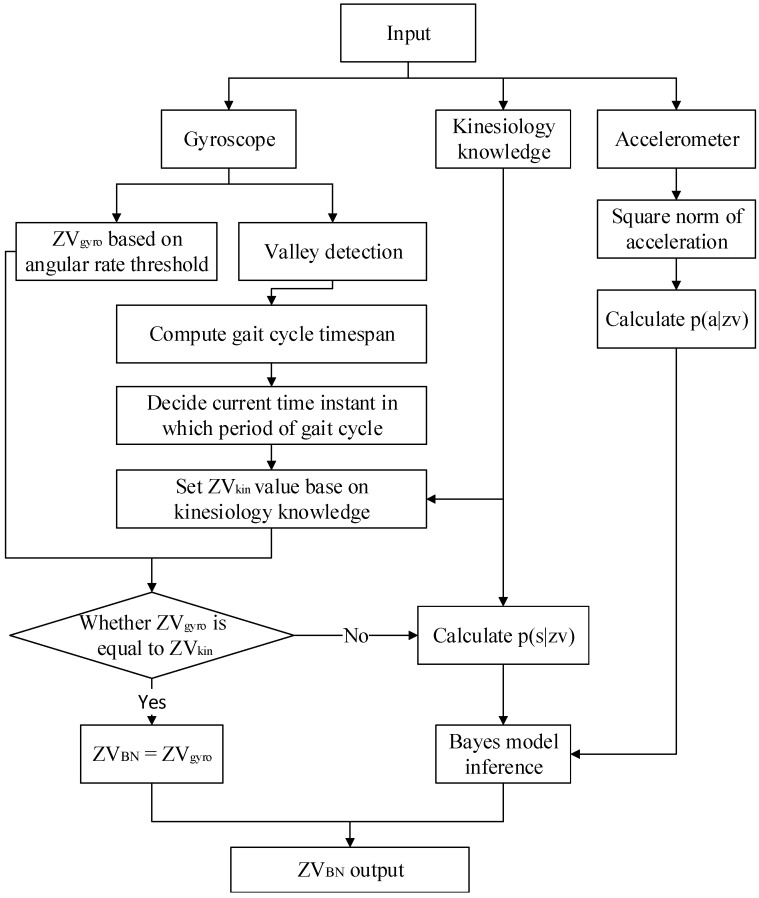
BN inference flow chart.

Figure 5 details the calculation flow of a ZV detector based on the BN inference method. The application of PINS normally runs on an embedded system, so the designed algorithm must be optimized to support resource-constrained devices. This method balances efficiency and accuracy. Firstly, the ZV_gyro_ is calculated by the ZV detector based on the angular rate threshold method. This method runs efficiently and works well in the swing and midstance periods. Secondly, according to kinesiology knowledge, ZV_kin_ is set as true in the midstance and propulsive period and false in the swing and contact period. Finally, if ZV_gyro_ is not equal to ZV_kin_, the proposed BN method is performed to infer whether it is zero velocity. Based on this strategy, the details of proposed ZV detector are as follows:

### 3.2. Zero Velocity Detector Based on Bayesian Model

**Figure 6:**
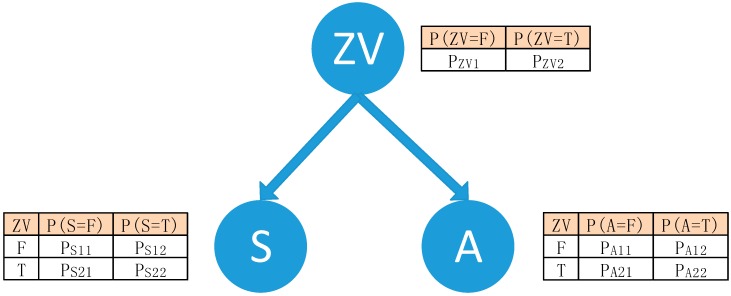
Bayes-based model of the ZV relationship.

To detect the ZV period more accurately, a Bayes-based model is proposed to make inferences. Figure 6 shows the structure of the model. The model has three nodes, namely, ZV represents the ZV state, S represents ZV_kin_ decided by the time instant in a single gait cycle and A represents ZV_acc_ decided by the magnitude of accelerometer measurements. Intuitively, S is set as true if the current time instant is in the midstance period and propulsive period, and false in the other periods. A is set as true according to Formula (1) if acceleration is between the thresholds. On this basis, the inference problem is to compute the most likely ZV state given the accelerometer measurements and the temporal information.

The target of the model is to detect the ZV period in a gait, so the model is binary, which “True” means the ZV state and “False” means non-ZV state. To define the parameters of this model, how to perform the inference is derived firstly. Bayesian Network is a model for probabilistic inference, the target value output by the model is inferred using the *maximum a posteriori* (MAP) equation:

(6) $$zv_{MAP}=argmax\;P\left(zv|s,a\right)$$

where *zv*, *s* and *a* are the values of *ZV*, *S* and *A* respectively. Rewritten using Bayes rule this yields:

(7) $$zv_{MAP}=argmax\frac{\;P\left(s,a|zv\right)P\left(zv\right)}{P\left(s,a\right)}=armax\;P(s,a|zv)P(zv)$$

In practice, the temporal information and accelerometer measurements are assumed conditionally independent. The Naive Bayes assumption is used to obtain:

(8) zv=argmax P(s|zv)P(a|zv)P(zv)

So the parameters learning problem becomes how to obtain the two conditional probabilities tables (CPT) for P(s|zv), P(a|zv), and the prior probability of P(zv).

### 3.3. Probability Analyze

**Figure 7:**
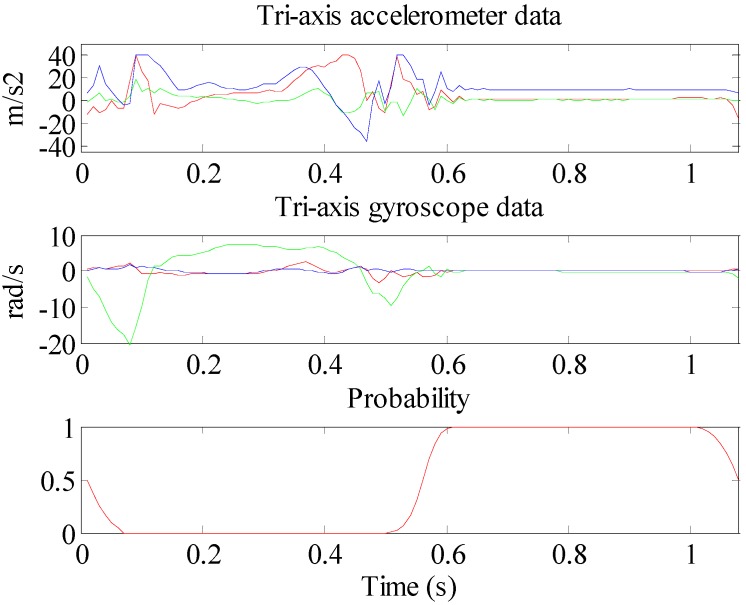
Measurements of accelerometer and gyroscope in one gait cycle, and CPD in measurement cycle.

Firstly, the P(s|zv) is analyzed. In theory, the two ZV boundaries are in the propulsive period and contact period. The proposed BN inference method mainly deals with these two periods. The probability of ZV in boundaries are assumed to obey a Gaussian distribution, and Cumulative Distribution Function (CDF) is used to describe the conditional probabilities tables of P(s|zv). The CPTs in propulsive and contact periods are given in Equations (9) and (10), respectively:

(9) $$P\left(s|zv\right)=\left[\begin{array}{cc} \frac{1}{2}[1+erf(\frac{t_{0}-t}{\sigma \sqrt{2}})] & 1-\frac{1}{2}[1+erf(\frac{t_{0}-t}{\sigma \sqrt{2}})]\\ 1-\frac{1}{2}[1+erf(\frac{t_{0}-t}{\sigma \sqrt{2}})] & \frac{1}{2}[1+erf(\frac{t_{0}-t}{\sigma \sqrt{2}})] \end{array}\right]$$

(10) $$P\left(s|zv\right)=\left[\begin{array}{cc} \frac{1}{2}[1+erf(\frac{t-(t_{0}+Tgait*0.548)}{\sigma \sqrt{2}})] & 1-\frac{1}{2}[1+erf(\frac{t-(t_{0}+Tgait*0.548)}{\sigma \sqrt{2}})]\\ 1-\frac{1}{2}[1+erf(\frac{t-(t_{0}+Tgait*0.548)}{\sigma \sqrt{2}})] & \frac{1}{2}[1+erf(\frac{t-(t_{0}+Tgait*0.548)}{\sigma \sqrt{2}})] \end{array}\right]\;$$

where $t_{0}$ is the end time of ZV period, namely the last ZV point in one gait calculated by Equation (3), $T_{gait}$ is a whole gait cycle timespan, 0.548 is the proportion of contact period and swing phase in a gait cycle (Figure 3), erf( ) is error function of normal distribution, σ is the covariance of Gaussian distribution. The setting of σ is very important, because the probability distribution in the transition between contact and midstance period should be smooth, and the same in the transition between propulsive and swing period. On the other hand, the probability during midstance period should be large because the measurements of IMU during this period will be variable when running. Large probability will make the ZV inference more reliable. 3 σ principle is used to calculate the value of σ:

(11) $$\sigma =C\times T_{gait}\times 0.01$$

where $T_{gait}$ is the timespan of a gait cycle, 0.01 is time coefficient because the minimal time unit is 10ms with discrete calculation, C is the constant, which takes the value as 2 and 3.5 in propulsive and contact period respectively through experiments. The evaluation of C value is shown in Section 5. The sketch map of CPD in measurement cycle is shown in the 3rd subplot of Figure 7.

Secondly, the relationship between ZV state and the measurements of the accelerometer P(a|zv) is discussed. Through the experiments, the Gaussian distribution is applied to describe the CPT of P(a|zv), based on the norm square of acceleration magnitude:

(12) $$P\left(a|zv\right)=\left[\begin{array}{cc} \frac{1}{2}[1+erf(\frac{norm(a_{n}-[0,0,g])}{\sigma \sqrt{2}})] & 1-\frac{1}{2}[1+erf(\frac{norm(a_{n}-[0,0,g])}{\sigma \sqrt{2}})]\\ 1-\frac{1}{2}[1+erf(\frac{norm(a_{n}-[0,0,g])}{\sigma \sqrt{2}})] & \frac{1}{2}[1+erf(\frac{norm(a_{n}-[0,0,g])}{\sigma \sqrt{2}})] \end{array}\right]$$

where norm() is the function of square norm, σ is the covariance of normal distribution which is set as 1. According to Equation (1), 1 σ principle is used to get the σ value, namely the probability of P(a|zv=true) should be above 0.683, when the value of $norm(a_{n}-\left[0,0,g\right])$ is in the range of [0, 1].

Finally, the setting of P(zv) is according to the kinesiology knowledge (Figure 3). The *zv* is set as true in the midstance period and propulsive period, and false in the contact period and swing phase. In practice, with the increase of speed, the proportion of ZV period will decrease. The experiments show that the P(zv) can work well in this Bayes model. The P(zv) is set as follows:

(13) $$\{\begin{array}{c} P\left(zv=true\right)\;=\;0.452\\ P\left(zv=false\right)=0.548 \end{array}$$

## 4. PINS Based on EKF

**Figure 8:**
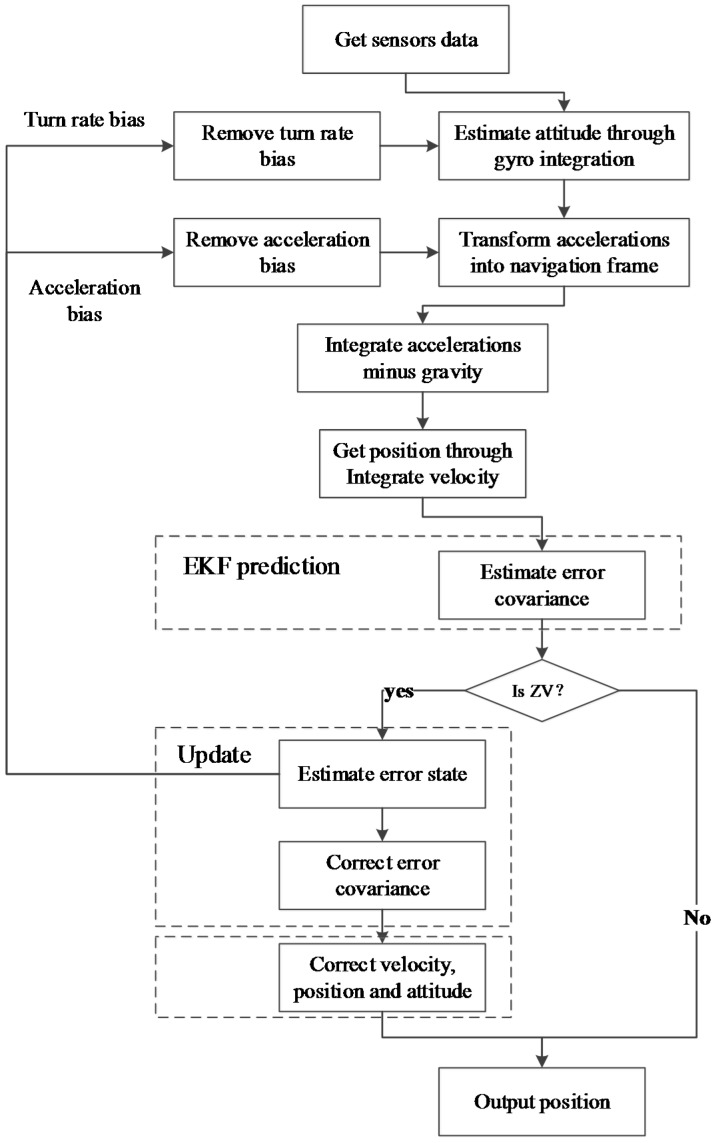
The EKF framework used for PINS.

During the ZV period detected by the proposed BN inference method, an EFK aided by ZUPT and ZARU is used to estimate the position error states of a pedestrian. Then the error states are fed back to the PINS for correcting the position. The EKF framework used for PINS is shown in Figure 8, and the details are described as follows:

### 4.1. Personal Inertial Navigation

The basic theory of PINS is to compute the IMU’s position and attitude in the navigation coordinate system from the IMU measurements in the body coordinate system, namely:

(14) $$a_{k}^{n}=\;C_{b_{k|k-1}}^{n}\cdot a_{k}^{b}$$

where $a_{k}^{b}$ is the noiseless acceleration in the body (b) coordinates at time k, $a_{k}^{n}$ is the noiseless acceleration in the navigation (n) coordinates, and $C_{b_{k|k-1}}^{n}$ is the rotation matrix which transforms vectors from the body coordinates to the navigation coordinates, given as follows:

(15) $$C_{b_{k|k-1}}^{n}=C_{b_{k-1|k-1}}^{n}\cdot \frac{2I_{3\times 3}+\delta \Omega_{k}\cdot ∆t}{2I_{3\times 3}-\delta \Omega_{k}\cdot ∆t}$$

where $C_{b_{k-1|k-1}}^{n}$ is the last rotation matrix which was corrected by the EKF, $C_{b_{k|k-1}}^{n}$ is updated with the incremental rotation term $\delta \Omega_{k}$, which is based on the bias-compensated gyroscopic readings. $\delta \Omega_{k}$ is the skew symmetric matrix for turn rates and represents the small angular increments in orientation:

(16) $$\delta \Omega_{k}=\left[\begin{array}{ccc} 0 & -\omega_{k}^{'b}(3) & \omega_{k}^{'b}(2)\\ \omega_{k}^{'b}(3) & 0 & -\omega_{k}^{'b}(1)\\ -\omega_{k}^{'b}(2) & \omega_{k}^{'b}(1) & 0 \end{array}\right]$$

Finally, the gravity is removed from the acceleration $a_{k}^{n}$. This gravity-free acceleration is integrated to obtain the velocity $v_{k}$. Then the position $r_{k}$ is gained by integrating this velocity. After that, the EKF is used to estimate the error states, which are updated with the IMU measurements in the ZV period. Thus the estimated errors could correct the previously computed attitude, velocity and position.

### 4.2. Perform ZUPT/ZARU Aided EKF

The error states EKF is performed during the ZV period detected by the proposed BN inference method. Because in this moment, the actual readings of accelerometer and gyroscope could be treated as error measurements, and the velocity and attitude are set as zero. This is the concept of ZUPT and ZART. The EKF error states vector contains 15 elements at time k:

(17) $$\delta x_{k|k}=\delta x_{k}={\left[\delta \varphi_{k},\delta \omega_{k}^{b},\delta r_{k},\delta v_{k},\delta a_{k}^{b}\right]}^{T}$$

where $\delta \omega^{b}$ and $\delta a^{b}$ represent the estimated biases for gyroscope and accelerometer respectively, δφ is the attitude errors, δr is the position errors and δv is the velocity errors in the navigation coordinates. Each of them has three-dimensional estimations. In the prediction stage, the linearized state transition model is:

(18) $$\delta x_{k|k-1}=F_{k}\delta x_{k-1|k-1}+w_{k-1}$$

where $\delta x_{k|k-1}$ is the predicted error states vector, $\delta x_{k-1|k-1}$ is the filtered error states vector at time k−1, $w_{k-1}$ is the process noise with covariance matrix **Q**, and $F_{k}$ is the 15 × 15 error states transition matrix:

(19) $$F_{k}=\left[\begin{array}{cc} \begin{array}{ccc} I_{3\times 3} & \Delta t\cdot C_{b_{k|k-1}}^{n} & 0_{3\times 3}\\ 0_{3\times 3} & I_{3\times 3} & 0_{3\times 3}\\ 0_{3\times 3} & 0_{3\times 3} & I_{3\times 3} \end{array} & \begin{array}{cc} 0_{3\times 3} & 0_{3\times 3}\\ 0_{3\times 3} & 0_{3\times 3}\\ \Delta t\cdot I_{3\times 3} & 0_{3\times 3} \end{array}\\ \begin{array}{ccc} -\Delta t\cdot S(a_{k}^{'n}) & 0_{3\times 3} & 0_{3\times 3}\\ 0_{3\times 3} & 0_{3\times 3} & 0_{3\times 3} \end{array} & \begin{array}{cc} I_{3\times 3} & \Delta t\cdot C_{b_{k|k-1}}^{n}\\ 0_{3\times 3} & I_{3\times 3} \end{array} \end{array}\right]$$

where Δt is the time step from the previous measurement, $a_{k}^{'n}$ is the bias-corrected acceleration in navigation frame transformed from $a_{k}^{'b}$ in body frame by Equation (13), $S(a_{k}^{'n})$ is the skew symmetric matrix using accelerations in the navigation coordinates to estimate the attitude of the sensor:

(20) $$S\left(a_{k}^{'n}\right)=\left[\begin{array}{ccc} 0 & -a_{z_{k}}^{n} & a_{y_{k}}^{n}\\ a_{z_{k}}^{n} & 0 & -a_{x_{k}}^{n}\\ -a_{y_{k}}^{n} & a_{x_{k}}^{n} & 0 \end{array}\right]$$

In the update stage, the EKF measurement model is:

(21) $$Z_{k}=H\delta x_{k|k}+n_{k}$$

where $z_{k}$ is the input error measurements, H is the measurement matrix and $n_{k}$ is the additive white zero-mean Gaussian noise with covariance matrix $R_{k}$. According to ZUPT/ZARU conception, the $Z_{k}$ and H are given by as follows:

(22) $$Z_{k}=\left[\delta \omega_{k}^{b}\;\delta v_{k}\right]=\left[\omega_{k}^{b}\;v_{k}\right]$$

(23) $$H=\left[\begin{array}{ccc} 0_{3\times 3} & I_{3\times 3} & 0_{3\times 3}\\ 0_{3\times 3} & 0_{3\times 3} & 0_{3\times 3} \end{array}\;\begin{array}{cc} 0_{3\times 3} & 0_{3\times 3}\\ I_{3\times 3} & 0_{3\times 3} \end{array}\right]$$

The estimated error states vector $\delta x_{k|k}$ is updated as follows:

(24) $$\delta x_{k|k}=\delta x_{k|k-1}+K_{k}\cdot [Z_{k}-H\delta x_{k|k-1}]$$

where $\delta x_{k|k-1}$ is the predicted error states vector. Noted that these filtered error states are used to compensate the attitude, velocity and position in PINS. The error states $\delta \omega_{k}^{b},\delta r_{k}\;and\;\delta v_{k}$ should be reset to zero, while $\delta a_{k}^{b}$ and $\delta \omega_{k}^{b}$ should be maintained over time. $K_{k}$ is the Kalman gain given by:

(25) $$K_{k}=P_{k|k-1}H^{T}{\left(HP_{k|k}H^{T}+R_{k}\right)}^{-1}$$

where $P_{k|k-1}$ is the estimation error covariance matrix based on measurements received at time k−1. The covariance matrix $P_{k|k}$ at time k is computed using the Kalman gain in the Joseph form equation:

(26) $$P_{k|k}=\left(I_{15\times 15}-K_{k}H\right)P_{k|k-1}{\left(I_{15\times 15}-K_{k}H\right)}^{T}+R_{k}$$

With the error estimator $\delta v_{k}$ and $\delta r_{k}$ obtained by Equation (24), the corrected velocity and position can be obtained by $v_{k}=v_{k}-\delta v_{k}$ and $r_{k}=r_{k}-\delta r_{k}$, respectively. Similarly, the attitude estimator can be corrected by Equation (15), in which the $\delta \Omega_{k}$ is composed of the error estimator $\delta \varphi_{k}^{b}$ with Equation (16). The estimated biases $\delta a_{k}^{b}$ and $\delta \omega_{k}^{b}$ are used to compensate the IMU measurements.

## 5. Implementation and Evaluation

Several tests have been performed to evaluate the performance of the algorithm. The application framework comprises an Android phone and an IMU with a Bluetooth module which is used to transfer the raw data to the Android unit (Figure 1). The IMU has 3-axis accelerometers and 3-axis gyroscopes. The choice of dynamic range of the IMU should balance the measurement resolution and the application demands. A wider dynamic range could provide more accurate altitude estimation [10], but sacrifice resolution. Moreover, the experiments show that the acceleration will exceed 80 m/s^2^ when running, so integrated analysis suggests that the range of the accelerometer should be set between ±160 m/s^2^. Similarly, the gyroscope is set between ±2000 deg/s. The sampling rate is set as 100 Hz, considering the computing resources of the Android platform.

The experiments are executed by different people indoors and outdoors. The scenarios include a floor with multiple rooms, multiple layers and outdoors. Since this application is mainly used in emergency rescue situations, ten physically fit volunteers with ages ranging from 20 to 35 took part in the experiments.

In this section, the proposed method is evaluated in two aspects. Firstly, the proposed ZV method is evaluated. The comparisons of ZV boundary and ZV false detections detected by different methods are shown. Secondly, the 3D Tracking Effect is evaluated. The volunteers travel different close paths to evaluate the horizontal error and upstairs to evaluate the vertical errors.

### 5.1. ZV Detector Evaluation

**Figure 9:**
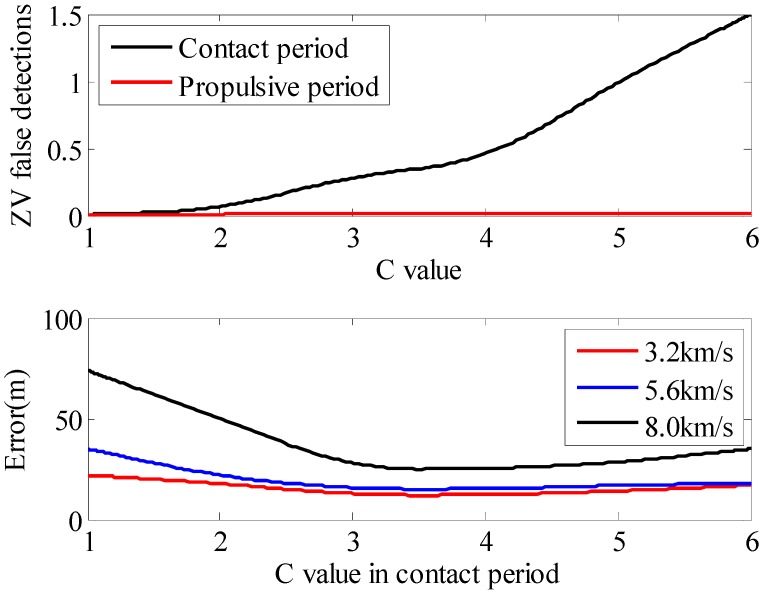
The average ZV detections in the contact period and propulsive period with different C values in Formula (11) and horizontal position errors with different C values in the contact period.

Firstly, In order to verify the Formula (11), the experiments analyzed the average ZV false detections in the contact period and propulsive period with different C values. The data is sampled at a speed of 5.6 km/h. The 1st subplot of Figure 9 shows that there exists more noise in the contact period. With the increase of C value, more false detections will occur. However, in the propulsive period, the signal feature has a sharp slope, so the number of false detections is small. This means that the C value in the propulsive period has a negligible effect on the algorithm. Thus, the experiment concentrates on the C value in the contact period. The 2nd subplot of Figure 9 shows the position error with different C values in the contact period. 

The experiment is performed over a 1 km rectangular closed path. According to the results, a small C value means that the probability distribution of P(s|zv) is steep, which will overlook the impact of P(a|zv). However, a large C value suggests that the BN ZV detector will degenerate into the combined condition method. The results show that the C value could be set as 3.5.

**Figure 10:**
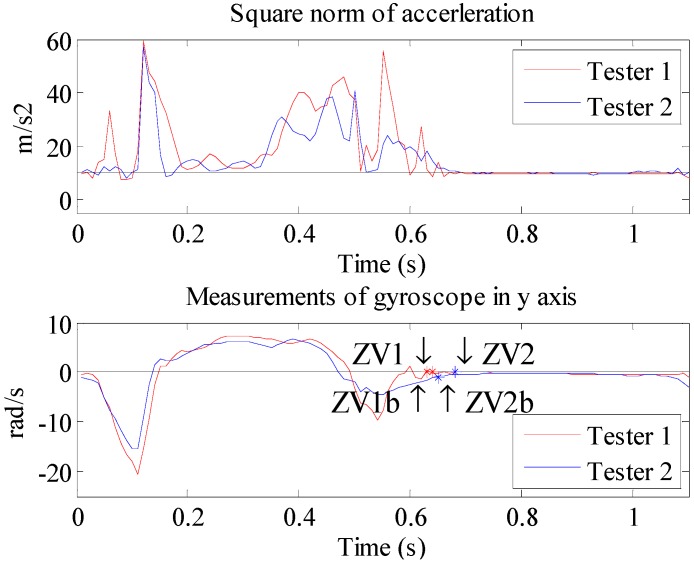
The square norm of acceleration and the gyroscope measurements in the *y* axis of two testers, and the comparison of ZV boundaries obtained by two methods

The comparison of the detected ZV boundary between the proposed method and the combined condition method is shown in Figure 10. Based on the data of two testers, the results suggest that the start of the ZV period determined by the proposed method is 0.01 s (from ZV1 to ZV1b) and 0.04 s (from ZV2 to ZV2b) earlier than the combined condition method, respectively. This is because the foot strikes the ground at the end of the contact period. During this process, more noise is created, which will cause the start time of the ZV period delay detected by the threshold method. Unfortunately, during this short delay time, the position error could increase rapidly.

**Figure 11:**
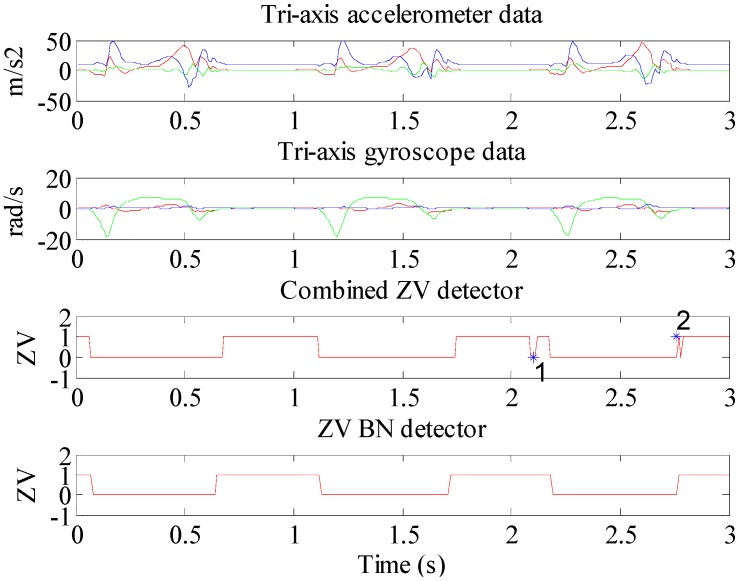
The comparison of ZV false detections between the combined condition method and the BN inference method.

In Figure 11, the top two subplots are the tri-axis accelerometer and tri-axis gyroscope measurements, respectively. The 3rd subplot is the ZV detected by the combined condition method with median filter, it should be noted that there still exist two ZV false detections, especially point 1 that occurs in the midstance period, which is impossible in practice. Point 2 is an ambiguous detection. The 4th subplot is the ZV result of the proposed method. This ZV result is clearer.

**Figure 12:**
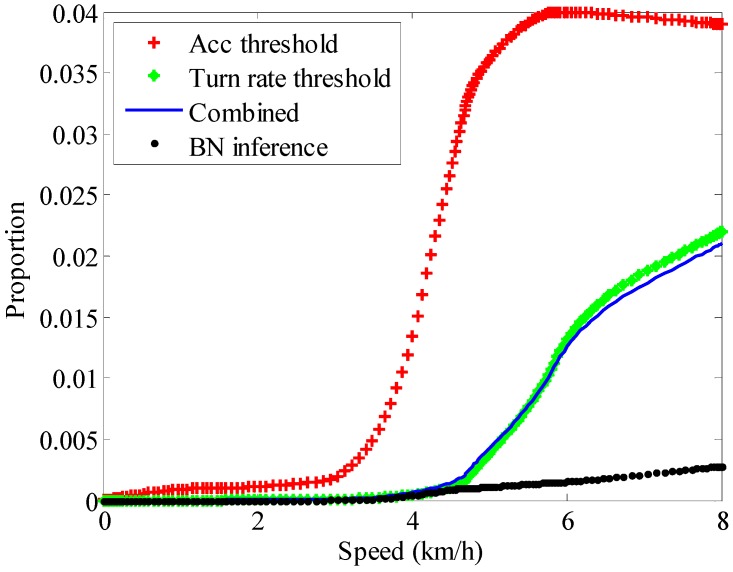
The proportion of ZV false detections in the total test duration with different methods at different speeds.

Through the experiments, Figure 12 shows the proportion of ZV false detections in the total test duration with different methods at different speeds. The experiments are performed at different speed of 1.8, 3.6, 5.6 and 8.0 km/h, respectively. It reveals that the combined method could work well during low speed movement, but when the speed increases, more noise will reduce the accuracy of the algorithm. Some researchers advise that the only angular rate threshold method can provide a relatively stable ZV detector, but in the test, it still could not achieve good performance at high walking speed. Most false detections occur during the transition from the contact period to the midstance period, while the BN inference method could use kinesiology knowledge to reduce the fuzziness of the ZV period to some extent. In short, with the increase of speed, the number of false detections will grow by all the methods, but the BN inference method could limit this growth effectively.

The proportion of detected ZV periods in one gait cycle obtained with different methods has been compared in Table 1. When the walking speed increases, more noise will occur. This is the reason why the dynamic range of the proportion by the combined condition method is wide. The range of the proposed method is more stable than the method under the combined conditions. On the other hand, when people are running, there exists only very tiny ZV period in one gait cycle based on the combined method, which will fail to locate pedestrians.

**Table 1:** The proportion of detected ZV period in one gait cycle by two methods.

Method	Combined Method (%)	Bayesian Network Inference (%)
**ZV in one gait**	18–46	32–47

### 5.2. 3D Tracking Effect Evaluation

**Figure 13:**
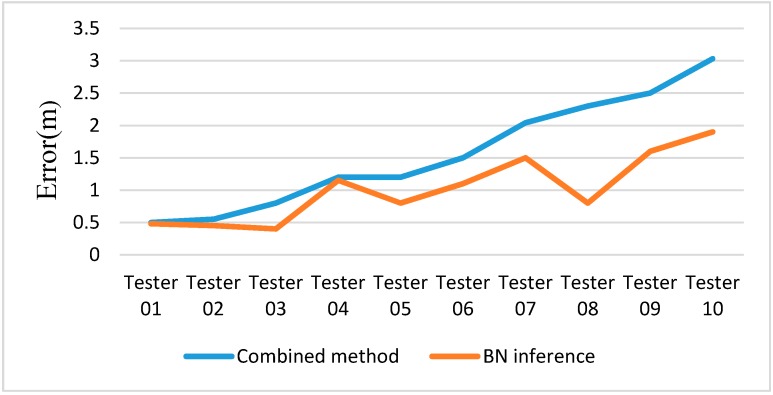
Experiment results of altitude error in a total distance of 100 m with ten testers at normal speed.

To evaluate the altitude accuracy of PINS based on the proposed ZV detector, experiments are performed on a horizontal floor. The accuracy of altitude is a tough problem in 3D tracking, because it is constrained for many reasons, including the dynamic range of the accelerometers, accelerometer sensor errors and the accuracy of the ZV detector, *etc.* The experiment reveals that the proposed method could improve the altitude accuracy, and reduce the altitude error by 40% on average (Figure 13). 

**Figure 14:**
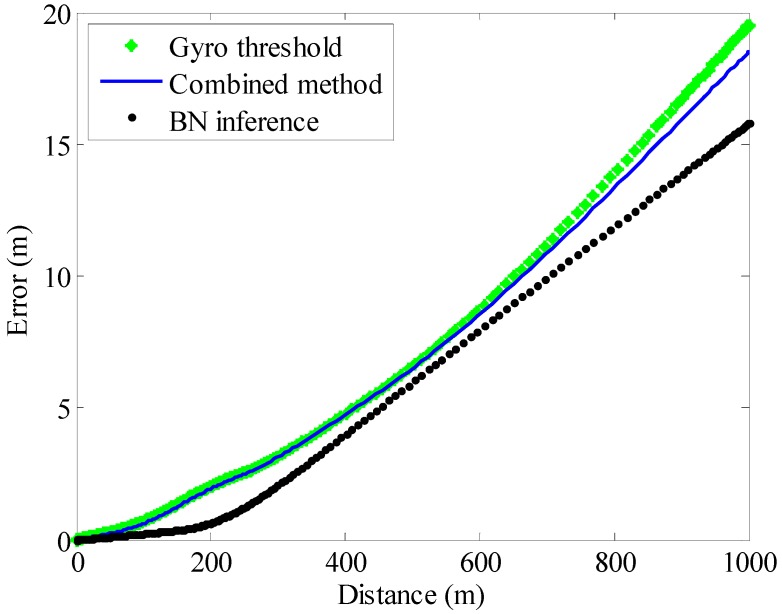
Average horizontal error by PINS/EKF based on different ZV detectors.

The entire system is tested in different scenarios. Figure 14 shows the average horizontal errors in the EKF/PINS algorithm based on different ZV detectors. The experiments are performed along a closed path at normal speed (5.6 km/h), and the difference between the start and end points is treated as the horizontal error. The tests’ rectangular paths are 100, 200, 300, 600 and 1000 m in length. The results show that the position error roughly increases linearly with distance, and the proposed ZV detector could also improve the tracking effect.

**Figure 15:**
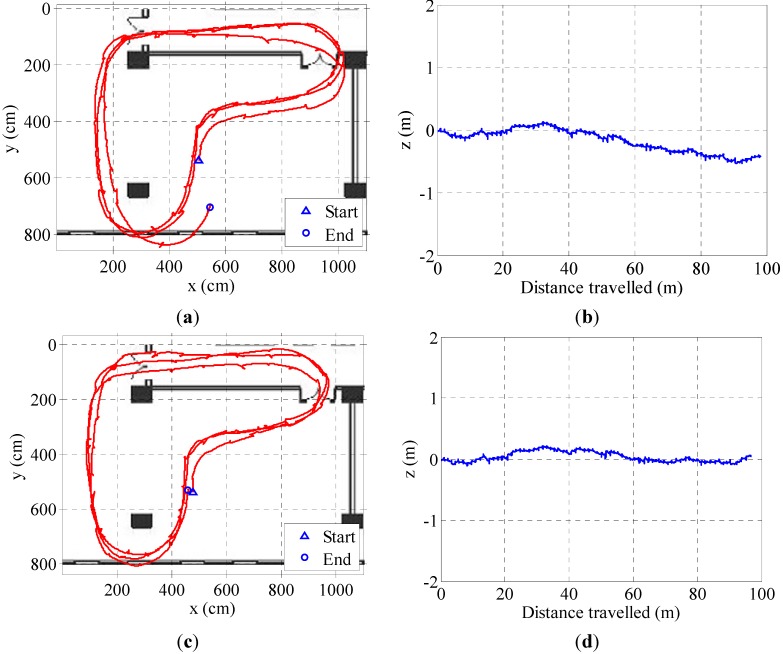
The comparison of the tracking results based on different ZV detectors. (**a**) Estimated 2D path based on the combined condition method; (**b**) Estimated altitude based on the combined condition method; (**c**) Estimated 2D path based on the BN method; (**d**) Estimated altitude based on the BN method.

**Figure 16:**
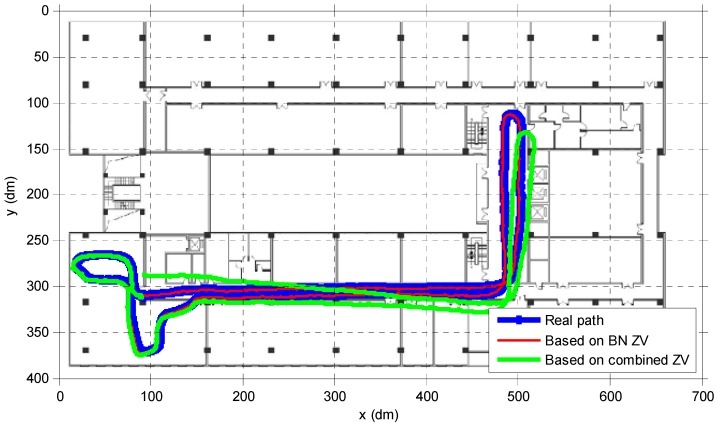
Estimated indoor 2D path.

**Figure 17:**
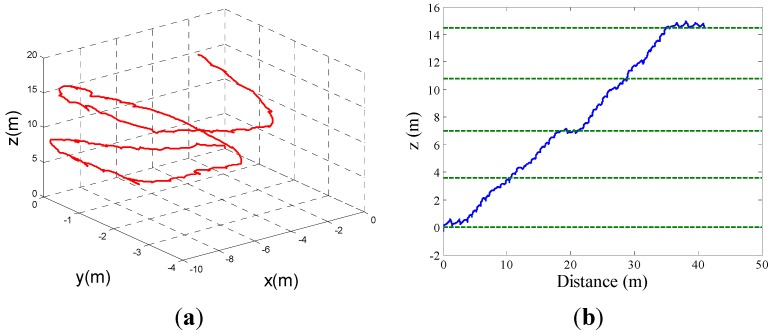
(**a**) Estimated 3D path for going upstairs; (**b**) Estimated altitude.

The experiments prove that the proposed method could compensate the altitude error better (Figure 15b,d). Meanwhile, an accurate ZV detector could also achieve better horizontal tracking effects. In Figure 16, the blue closed path is the real walking path. With the same EKF parameters, the red path based on ZV detector by BN inference method could fit the real path well, while based on the combined condition ZV detector, the green tracking path deviates from the real path. Figure 17 shows the 3D map and altitude map of going upstairs, the result shows a good altitude tracking effect by the EKF/PINS based on the ZV period detected by the BN inference method.

The proposed method to detect the ZV period is robust. To get a better position effect, it is essential to adjust the EKF parameters based on sensor characteristics and the different movement patterns. The experiments show that a different R value in EKF will affect the result. Intuitively, the increase of R will lead to over-steering, and *vice versa*. This is because when R is big, namely the uncertainty of velocity and angular rate is big, and then the EKF will compensate more. However, the influence of R value on the result is limited because the EKF will converge. In the future, some landmarks could be used to decide whether the tracking is over-steering or under-steering, and adjust R value adaptively. On the other hand, more aided information can be introduced to calibrate the position errors, like maps, RF markers and so on.

## 6. Conclusions

This paper proposes a robust zero velocity detector algorithm used in personal navigation systems based on a foot-mounted IMU. The ZV detector by Bayesian Network inference method is introduced, with the measurements of inertial sensors and kinesiology knowledge. The algorithm uses the angular rate signal features to accurately compute gait cycles and get the start time of a gait cycle. The experiments show that the BN inference method could reduce the boundary fuzziness of the ZV period and effectively remove the ZV false detections in tough application situations. With the detected ZV period, an EKF/PINS aided by ZUPT/ZARU achieves a good 3D tracking effect.
